# Draft Genome Sequences of Two South African *Bacillus anthracis* Strains

**DOI:** 10.1128/genomeA.01313-15

**Published:** 2015-11-19

**Authors:** Kgaugelo E. Lekota, Joseph Mafofo, Evelyn Madoroba, Jasper Rees, Henriette van Heerden, Farai C. Muchadeyi

**Affiliations:** a Biotechnology Platform, Agricultural Research Council, Onderstepoort, South Africa; b Department of Veterinary Tropical Diseases, University of Pretoria, Onderstepoort, South Africa; c Agricultural Research Council, Onderstepoort Veterinary Institute, Onderstepoort, South Africa; d University of South Africa College of Agriculture and Environmental Sciences, Florida Campus, Florida, South Africa

## Abstract

*Bacillus anthracis* is a Gram-positive bacterium that causes anthrax, mainly in herbivores through exotoxins and capsule produced on plasmids, pXO1 and pXO2. This paper compares the whole-genome sequences of two *B. anthracis* strains from an endemic region and a sporadic outbreak in South Africa. Sequencing was done using next-generation sequencing technologies.

## GENOME ANNOUNCEMENT

Anthrax is a zoonotic bacterial disease caused by the spore-forming soil-borne bacterium *Bacillus anthracis*. Fully virulent wild-type strains of *B. anthracis* carry two large plasmids, pX01 and pX02 (182 and 96 kb, respectively), which harbor the virulence factors (1). Anthrax occurs endemically in the Northern Cape region (NCP) and Kruger National Park (KNP), but sporadic outbreaks occur in livestock and wildlife throughout the country.

In this study, two strains were sequenced, 3631_1C, isolated from a kudu (*Tragelaphus strepsiceros*) in the NCP during the 2009 outbreak in Klipfontein, and 20SD, isolated from a sporadic outbreak in sheep (*Ovis aries*) in the Standerton area, Mpumalanga Province, South Africa. The genotyping method, multilocus variable-number tandem-repeat analysis (MLVA) (2, 3), clustered both *B. anthracis* strains in the A clade.

The sequencing libraries were generated using the Nextera XT DNA sample preparation kit (Illumina) and sequenced on the Illumina HiScan SQ (Illumina) instrument. A total of approximately 2.0 and 3.0 million 100-bp paired-end reads were generated for 20SD and 3631_1C, respectively, and analyzed using CLC Genomics Workbench version 7. The genome assemblies of the strains were aligned to *B. anthracis* Ames Ancestor (GenBank accession numbers AE017334, AE017336, and AE017335) (4) using the Burrows-Wheeler Aligner (BWA) (5). Approximately 99% of the reads generated for both the 20SD and 3631_1C strains mapped to the reference chromosome and the plasmids. The sequence data for 20SD had 30-fold coverage of the chromosome, 98-fold coverage of pXO1, and 48-fold coverage of the pXO2. The sequence data for the 3631_1C strain gave 53-fold coverage of the chromosome, 127-fold coverage of plasmid pXO1, and lacked pXO2.

The sequence data were also used for *de novo* assembly of the two strains using CLC Genomics Workbench version 7. The assembled contigs were aligned using BLASTn (6) using *B. anthracis* Ames ancestor as a reference. The assembled contigs were ordered using Mauve (7) and ABACAS (8) to check for consistency in overlapping contigs. The assembled genomes comprise 52 and 45 contigs for strains 20SD and 3631_1C, respectively, with 50 and 44 contigs mapping to the chromosomes for strains 20SD and 3631_1C, respectively, while the plasmids, pXO1 and pXO2, assembled into 1 contig each.

The draft genomes were annotated using the RAST (9). Strain 3631_1C gave a total of 5,680 coding sequences (CDSs) on the chromosome, 204 CDSs on plasmid pXO1, and a total of 64 tRNAs, whereas pXO1 and pXO2 of strain 20SD produced 201 and 117 CDSs, respectively, 5645 CDSs on the chromosome and a total of 49 tRNAs. The draft genome sequences for *B. anthracis* 20SD and 3631_1C were approximately 5.44 and 5.35 Mb, respectively. Both strains yielded an average G+C content of 35%.

Previous studies have shown that *B. anthracis* strains*,* especially those isolated from the soil and/or after long-term culturing, usually lack one or both plasmids (10, 11). This was also observed in this study in strain 3631_1C, whereas strain 20SD was a fully virulent *B. anthracis* strain.

### Nucleotide sequence accession numbers.

The draft genome sequences of the *B. anthracis* 20SD and 3631_1C strains are available in GenBank under the accession numbers LGCC00000000 and LGCD00000000, respectively.
